# Creation of an immunodeficient HLA-transgenic mouse (HUMAMICE) and functional validation of human immunity after transfer of HLA-matched human cells

**DOI:** 10.1371/journal.pone.0173754

**Published:** 2017-04-11

**Authors:** Yang Zeng, Bingrun Liu, Marie-Thérèse Rubio, Xinyue Wang, David M. Ojcius, Ruoping Tang, Antoine Durrbach, Zhitao Ru, Yusen Zhou, Yu-Chun Lone

**Affiliations:** 1 State Key Laboratory of Pathogen and Biosecurity, Beijing Institute of Microbiology and Epidemiology, Beijing, China; 2 INSERM U1197 (ex U1014), Hospital Paul Brousse, University of Paris-Sud, Université Paris-Saclay, Villejuif, France; 3 Service d'Hématologie de médecine interne, Hôpital Brabois, CHRU Nancy, Vandoeuvre les Nancy, France. And CNRS UMR 7365, IMoPa, Biopole de l’Université de Lorraine, Vandoeuvre les Nancy, France; 4 Department of Biomedical Sciences, University of the Pacific, Arthur Dugoni School of Dentistry, San Francisco, CA, United States of America; 5 Tumorothèque du service d’Hématologie de l’Hôpital Saint-Antoine. Assistance Publique-Hôpitaux de Paris (AP-HP), Paris, France; 6 IFRNT, Nephrology department, Le Kremlin Bicetre, Universite Paris-Sud, France; McMaster University, CANADA

## Abstract

Research on human immunology has been hindered by the lack of optimal small animal models, given that the protective immune responses of human and non-human species show significant differences. However, due to ethical constraints[1] and the high cost of clinical trials, it is urgent to improve the current animal models that can mimic faithfully human physiology, particularly the human immune system (HIS). HIS mice had been generated recently by engrafting human hematopoietic stem cells (hHSCs) or human peripheral mononuclear cells (hPBMCs) into highly immuno-deficient mice such as NSG, NOG or NRG mice. However, a major experimental drawback for studies using these models is the rapid onset of Graft-*versus*-Host Disease (GvHD). In the present study, we overcome this limitation by generating new immuno-deficient mice named "HUMAMICE" (HLA-A2^+/+^/DR1^+/+^/H-2-β_2_m^-/-^/IAβ^-/-^/Rag2^-/-^/IL2rγ^-/-^/Perf^-/-^ mice), which expressed human HLA molecules instead of mouse MHC molecules (H-2), and whose immuno-deficient status was reversed by transferring functional HLA-matched PBMCs thus producing mice with an immuno-competent status with a functional human immune system. We showed that in this HLA-matched context, the hPBMC-transfer led to high lymphocytes engraftment rates without GvHD over three months in this novel mouse model. Furthermore, to evaluate the utility of the hPBMC-HUMAMICE, we immunized them with commercial vaccine of Hepatitis B virus (HBsAg, Hepvac@) which resulted in robust and reproducible production of high levels of HBsAg-specific antibodies, implying that both transferred T and B lymphocytes were functional in HUMAMICE. These responses are comparable to those observed in human clinical trials with this identical vaccine. In conclusion, these findings indicated that the HLA-matched-hPBMC-HUMAMICE represents a promising model for dissecting human immune responses in various human diseases, including infectious diseases, cancers and tumors, and to facilitate the development of novel vaccines and cellular therapies.

## Introduction

Mice functionally engrafted with human hematopoietic cells (hHSCs) may represent a valuable preclinical tool for basic and applied investigations of the human immune system[2]. The current reference models for such humanized mice involved grafting a functional human immune system into the immuno-deficient mice, for example, NSG [3], NOG, NRG, Rag2^-/-^ [4] and IL2rγ^-/-^ mice [5–7] (these highly immuno-deficient mice lack almost the entire mouse-derived immune system, including murine B cells, T cells, and natural killer cells). These models have certain advantages for fostering the survival and expansion of human donor cells, but also have several limitations and drawbacks[8,9]. However, the maintenance of HLA compatibility is necessary for ensuring the proper function of a normal human immune system in a murine genetic background. Indeed, after hPBMC transferred, a constant and rapid onset of xenogenic Graft-*versus*-Host Disease (xeno-GvHD[10]) was observed in all hPBMC-humanized mice. This complication caused researchers to avoid these models for studies of the human immune system [11], in particular, to explore responses against infections such as human-tropic infectious diseases (caused by Epstein–Barr virus and HIV[12]). Consequently, the models are not used in the development of vaccines and cell or gene therapies[13].

Nonetheless, these PBMC-humanized mice are still an excellent xeno-GvHD model for evaluating therapeutic strategies that could interfere with xeno-GvHD development. Unfortunately, while the transfer of hPBMC leads to high lymphocytes engraftment rates, the time-frame for experimental intervention and analysis is somewhat limited, because of the rapid development of xenogenic GvHD.

The principal host components responsible for triggering GvHD are the xenogenic mouse MHC (H-2) class I and class II molecules. Studies with NSG mice lacking H-2 class I (β_2_m^null^) or H-2 class II (IAβ^null^) showed that the deletion of H-2 class I and II molecules could delay the occurrence of the diseases significantly compared with WT NSG mice, but could not abrogate it completely[14,15]. By contrast, H-2 class I-deficient NSG mice were relatively resistant to the development of xeno-GvHD. These data indicated that both donors’ CD8^+^ and CD4^+^ T cells contribute significantly to development of xenogeneic GvHD and revealed the critical role of H-2 class I and class II molecules in the development of xeno-GvHD in mice. Furthermore, in contrast to the repopulation of NRG mice with xenogeneic human DQ8-PBMCs which resulted in rapid induction of xeno-GvHD and poor survival rate of the animals, the transfer of HLA-class II-matched human DQ8-PBMCs into NRG IAβ^–/–^DQ8 transgenic recipients (lacking the expression of murine H-2 class II molecules, and expressing a transgenic HLA-DQ8 molecule) resulted in a milder form of xeno-GvHD, such that the mice survived significantly longer than other previous mouse models. These data showed that HLA compatibility between donors and recipients is a requirement for ensuring a proper function of human immune systems in mice without the emergence of graft rejection.

Previously, we have developed an HLA class I and class II transgenic mouse (Sure-L1 mice) (HLA-A2^+/+^/DR1^+/+^/H-2-β_2_m^-/-^/IAβ^-/-^) which lacked both murine H-2 class I and II molecules, and instead expressed HLA-A2 and HLA-DR1 molecules[16]. In this humanized mouse model, murine T cells could only mount HLA-restricted responses without H-2-restricted responses, demonstrating that the expression of both transgenic HLA class I and II molecules could result in proper thymic education and differentiation of developing T lymphocytes.

In this study, we describe a novel mouse strain derived from the Sure-L1 mouse (HLA-A2^+/+^/DR1^+/+^/H-2-β_2_m^-/-^/IAβ^-/-^), in which we additionally depleted the murine Rag 2 allele, the interleukin 2 receptor gamma chain allele (IL2rγ) and the perforin allele (Perf), leading to the generation of immuno-deficient mice (HLA-A2^+/+^/DR1^+/+^/H-2-β_2_m^-/-^/IAβ^-/-^/Rag2^-/-^/IL2rγ^-/-^/Perf^-/-^ mice) which named HUMAMICE.

Furthermore, we confirmed that the transfer of HLA-matched hPBMCs into this HUMAMICE can generate a functional human immune system without signs compatible with onset of Graft-versus-Host Disease (GvHD) including the pathological symptoms like weight loss, diarrhea, alopecia or the immunological signs like infiltration of activated human lymphocytes in liver, skin, or intestine.

Therefore, the HLA-matched-hPBMC-HUMAMICE model opens the possibility of studying not only normal human immune-hematopoietic development but also human disease pathogenesis for a broad range of biomedical applications.

## Methods

### Ethics statement

Experimental procedures were approved by the Animal Care Committee of the University Paris-Sud Network, and carried out in accordance with Animal Care Guidelines. Human PBMCs were collected from healthy volunteers (with written informed consent, that these documents are recorded in tumorotheque of hopital saint Antoine) in Hospital Saint Antoine and in compliance with French and European regulations. Tumorotheque of hopital saint Antoine 's review board specifically approved the study regarding the collection of Human PBMCs valid until June 30th, 2016

### Mice

HUMAMICE (HLA-A2^+/+^/DR1^+/+^/H-2-β_2_m^-/-^/IAβ^-/-^/Rag2^-/-^/IL2rγ^-/-^/Perf^-/-^ mice) were generated on the background of Sure-L1 mice (HLA-A2^+/+^/DR1^+/+^/β_2_m^-/-^/IAβ^-/-^) to homozygosity. The construction of SURE-L1 mice was fully described previously^16^. In brief, we established the homozygous SURE-L1 mice by intercrossing the HLA-A2.1 (HHD)-transgenic H-2 class I-KO (β_2_m^-/-^) mice (genetic background of C57BL/6 backcrossed 9 generation) with HLA-DR1-transgenic H-2 class II-KO (IAβ^-/-^) mice (genetic background of C57BL/6 backcrossed for 7 generation). HUMAMICE was produced by several intercrossings between Sure-L1 mice with Rag2^^null^^ mice, IL2rγ^^null^^ mice and Perf^^null^^ mice. The obtained final homozygote HUMAMICE (HLA-A2^+/+^/DR1^+/+^/H-2-β_2_m^-/-^/IAβ^-/-^/Rag2^-/-^/IL2rγ^-/-^/Perf^-/-^) were checked by both specific PCR molecular genotyping and FACS analysis after specific immune-labelling (data not shown).

HUMAMICE were bred and housed under specific pathogen-free (SPF) conditions in isolators in the animal facility at the TAAM Orléans center, and using sterile techniques and micro-isolator caging. Experimental procedures were approved by the Animal Care Committee of the University Paris-Sud Network, and carried out in accordance with Animal Care Guidelines. Six to eight week-old female mice were used as transplantation recipients. The endpoint for the survival study was set when recipient mice looked clinically ill or lost more than 15% of body weight, and mice were humanely sacrificed by cervical dislocation under general anesthetic or CO_2_.

### Genotype identification of HUMAMICE

The transgenic of HLA-A2 and HLA-DR1, as well as the knockout of H-2-β_2_m, H-2-IAβ, Rag2, IL2rγ and Perf were identified by specific PCR test. Murine genomic DNA was extracted as described previously[17]. The immuno-deficiency characteristic of HUMAMICE was checked by flow cytometry, while the immuno-competent C57BL/6 mice were chosen as the controls. Splenocytes were labelled with mCD3-PE and mCD19-FITC, mCD4-APC and mCD8-PE-Cy5.

### The identification of the immuno-deficiency of HUMAMICE

To confirm the immuno-deficiency status of HUMAMICE, we engrafted the subcutaneously the human RAMOS tumor cells (10^7^ cells) into HUMAMICE and immuno-competent parental Sure-L1 mice. Furthermore, to verify that the inability of HUMAMICE to reject RAMOS cells is due to lack of the mouse-derived immune system, 30 days after the injection of RAMOS cells, we transferred total splenocytes, purified CD8^+^ T lymphocytes or other lymphocytes derived from immuno-competent HLA-A2/DR1 mice which had rejected RAMOS cells into HUMAMICE carrying a solid tumor. Moreover, we surveyed and measured the volume of tumors constantly.

### Collection and purification of human PBMC

Human PBMCs were collected from healthy volunteers in Hospital Saint Antoine and compliance with French and European regulations. PBMCs were purified by Ficoll-Hypaquedensity centrifugation and suspended in PBS.

### HLA genotyping

The HLA-A02*01 (HLA-A2) positive individuals were identified by flow cytometry. And the DNA was extracted from 1×10^6^donor hPBMCs using the DNeasy Blood & Tissue Kit (Qiagen). The HLA-DRB1-01 (HLA-DR1) positive individuals were identified by specific polymerase chain reaction (PCR) using Olerup SSP DR low resolution and high solution SSP DRB1:01 kit following the instructions.

### Xenogenic hPBMC transplantation

Female 6- to 8-week-old HUMAMICE were irradiated with 3 Gy using a cobalt radiation source shortly the day before cell transfer (Precision X-RAD 320 Biological Irradiator) and were followed given by intravenous injection of 10^7^ hPBMC cells with a total volume of 100 μL per recipient mouse. After transplantation, mice were given sterile water containing prophylactic neomycin sulfate. And mice were monitored for weight and clinical symptoms twice per week. Weight loss of > 15% of original starting weight is a sign of GvHD development and necessitates euthanasia of the PBMC-engrafted mice.

Two independent experiments with two different donors were performed using a group of 5 or 4 mice. For HLA-A2^+^/DR1^+^ donor A, we transferred with 5 mice. Three HUMAMICE were analyzed at five weeks post hPBMC transfer, and two were sacrificed at 8 weeks post hPBMC transfer for FACS analysis. For HLA-A2^+^/DR1^+^ donor B, we transferred with 4 mice. HUMAMICE (2 at 5 weeks post hPBMC transfer, 1 mouse at 8 weeks and 1 mouse at 12 weeks post hPBMC transfer) were sacrificed for FACS analysis.

### Immunization of HUMAMICE

Fourteen days after the transplantation of hPBMCs, HUMAMICE received three intramuscular (i.m.) injections of recombinant HBV vaccine (10 μg) (Pasteur Institute) in a total injected volume of 100 μL. Each immunization was separated by an interval of 14 days. Sera were collected both before and after each immunization boost for serological analysis. Mice were sacrificed 10 days after the final boost.

### Flow cytometry

To detect human cells engrafted in HUMAMICE, multi-colors cytometric analysis was performed using *BD* LSR Fortessa, according to the manufacturer’s protocol but with a minor modification. HUMAMICE were sacrificed and the spleens were removed at three different times after hPBMCs were transplanted. Splenocytes were collected and lysed by ACK lysis buffer, counted and incubated with an appropriate volume of antibodies for 1 hour at 4°C and then subjected to flow cytometry analysis.

Commonly used antibodies included: Anti-Human CD45 FITC, Clone: 2D1, (ebioscience9011‐9459); isotype control, Mouse IgG1 K Isotype Control (ebioscience, FITC 11–4714) ; Anti-Mouse CD45 eFluor^®^ 450 (ebioscience, 48-0451-82) ; isotype control, Mouse IgG2b K Isotype Control eFluor^®^ 450 (ebioscience, 48-4732-82) ; Anti-Human CD3 APC-eFluor^®^ 780, Clone: UCHT1, (ebioscience, 47–0038) ; Mouse IgG1 K Isotype Control APC- eFluor^®^ 780 (ebioscience, 47–4714) ; Anti-Human CD4 PerCP-Cyanine5.5, Clone: OKT4 (OKT-4), (ebioscience, 45–0048); isotype control, Mouse IgG2b K Isotype Control PerCP-Cyanine5.5 (cat. 45–4732) ; Anti-Human CD8a PE-Cyanine7, Clone: SK1, (ebioscience, 25–0087); isotype control, Mouse IgG1 K Isotype Control PE-Cyanine7 (ebioscience, 25–4714) ; Anti-Human CD19 APC, Clone: 2H7, (ebioscience, 17–0209); isotype control, Mouse IgG1 K Isotype Control APC (ebioscience, 17–4714).

### Measurement of serum antibodies by ELISA

The serum levels of antibodies specific for the commercial HBsAg vaccine in immunized HUMAMICE were measured by ELISA test. The plates coated with the HBsAg vaccine were blocked with PBS supplemented with 0.1% Tween20 and 10% FCS and then washed three times. After the addition of mouse serum for 1 h, the plates were washed again, and bound antibodies were detected with Anti-Human IgG (whole molecule)-Peroxidase antibody produced in rabbit (SIGMA-ALDRICH, A8792) and Anti-Human IgM (μ-chain specific)-Peroxidase antibody produced in goat-affinity isolated antibody (SIGMA-ALDRICH, A6907). Absorbance was then measured at 450_nm_ in a plate reader. The antibody titers (the mean of at least three determinations) were calculated using the serial end-point dilution method.

### Statistical analysis

Each experiment was repeated at least three times, and data were expressed as the mean ± SEM. The means or geometric means of multiple groups were compared using Student’s t-test. All statistical analysis was performed using GraphPad Prism version 5.0. A P value<0.05 was considered statistically significant.

## Results

### Generation and characterization of HUMAMICE

Based on the background of Sure-L1 mice (HLA-A2^+/+^/DR1^+/+^/H-2-β_2_m^-/-^/IAβ^-/-^ mice), we generated the humanized mice “HUMAMICE”. This was achieved by several rounds of intercrossing between Sure-L1 mice with Rag2^-/-^ mice, IL2rγ^-/-^ mice and Perf^-/-^ mice. The resulting homozygote HUMAMICE (HLA-A2^+/+^/DR1^+/+^/H-2-β_2_m^-/-^/IAβ^-/-^/Rag2^-/-^/IL2rγ^-/-^/Perf^-/-^) were determined by both specific PCR molecular genotyping and FACS analysis after specific immune-labelling (data not shown).

**Fig 1:**
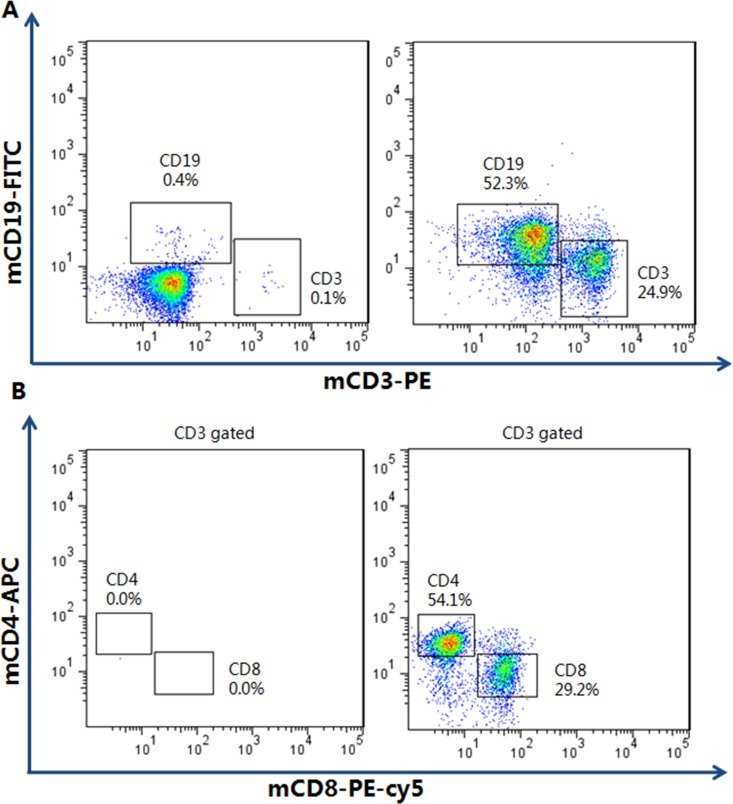
Flow cytometry analysis of immuno-deficient HUMAMICE. Comparative cell analysis by flow cytometry between immuno-deficient HUMAMICE (HLA-A2^+/+^/DR1^+/+^/H-2-β_2_m^-/-^/IAβ^-/-^/Rag2^-/-^/IL2rγ^-/-^/Perf^-/-^) and immuno-competent C57BL/6 mice. The left panel shows the analysis of immuno-deficient HUMAMICE and the right panel was the immuno-competent C57BL/6 mice. (A) Samples in Fig 1A were double-labelled with mCD3-PE and mCD19-FITC. (B) Samples in Fig 1B were labelled with mCD4-APC and mCD8-PE-Cy5.

We confirmed the immuno-deficiency characteristics of HUMAMICE by flow cytometry. Highly immuno-deficient mice such as *NOG/NSG* mice are characterized by the lack of almost the entire mouse-derived immune system, including murine B cells, T cells, and NK cells. Comparative cell analysis by flow cytometry between splenocytes from immuno-deficient HUMAMICE and immuno-competent C57BL/6 mice were shown in Fig 1. Representative immuno-competent C57BL/6 mice expressed 24.9% mCD3^+^ T cells and 52.3% mCD19^+^ B cells; while only a residual population (<0.5%) for both mCD3^+^ T cells and mCD19^+^ B cells was found in immuno-deficient HUMAMICE (Fig 1A). The ratio of mCD4^+^/mCD8^+^ T cells in C57BL/6 mice was 1.85 (54.1%/29.2%) (Fig 1B, right) and there were neither CD4^+^ nor CD8^+^ T cells in HUMAMICE (Fig 1B left). The absence of murine T cells (CD3^+^CD4^+^ T cells and CD3^+^CD8^+^ T cells) and B cells (CD19^+^CD20^+^) is an important requirement for the immuno-deficient status of these mice. Table 1 summarizes the results for other cell populations after the hPBMC engraftment. We noted, in particular, the absence of NK cells in HUMAMICE (0%) compared with immuno-competent C57BL/6 mice (1.9% and 2.4%). The consequence of this lack of almost the entire mouse-derived immune system is the sign of severe immune-deficiency characterized by the inability to reject foreign cells.

**Table 1:** Summary of comparative cell analysis was implemented by flow cytometry between immuno-deficient HUMAMICE and immuno-competent C57BL/6 mice.

Cellular type	HUMAMICE-1	HUMAMICE-2	C57BL/6 mice-1	C57BL/6 mice-2
mCD45^+^	44.9%	32.1%	88.8%	95%
mCD3^+^mCD4^+^	0.4%	0.2%	13.5%	27.5%
mCD3^+^mCD8^+^	0.4%	0.5%	7.3%	17.1%
mCD19^+^mCD20^+^	0.1%	0%	52.3%	32%
mCD19^-^mCD20^+^	0.1%	0.7%	2.4%	2.5%
NKp46^+^NK1.1^+^	0%	0%	1.9%	2.4%
Ter119^+^	18.2%	30.8%	5%	5.7%
CD11b^+^	9.4%	8.5%	6.4%	8.5%

The populations mCD45^+^; mCD3^+^mCD4^+^; mCD3^+^mCD8^+^; mCD19^+^mCD20^+^; mCD19^-^mCD20^+^; NKp46^+^NK1.1^+^; Ter119^+^; are CD11b^+^are presented.

### Confirmation of the immuno-deficiency of HUMAMICE

**Fig 2:**
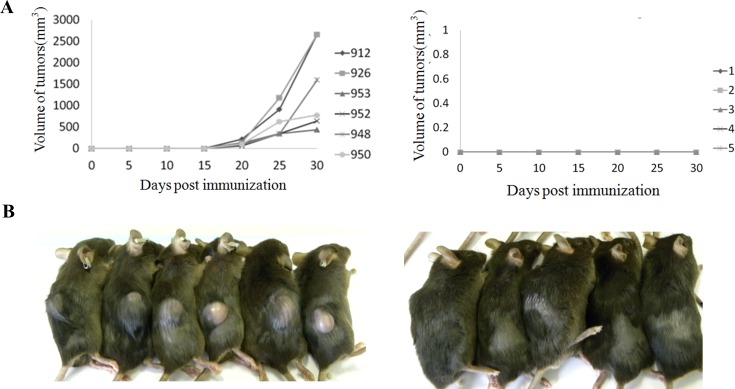
The tumor growth kinetics in immuno-deficient HUMAMICE and immuno-competent Sure-L1 mice after subcutaneous injection of RAMOS tumor cells. (A) Comparative tumor growth in six immuno-deficient HUMAMICE (HLA-A2^+/+^/DR1^+/+^/H-2-β_2_m^-/-^/IAβ^-/-^/Rag2^-/-^/IL2rγ^-/-^/Perf^-/-^) and five immuno-competent Sure-L1 mice (HLA-A2^+/+^/DR1^+/+^/H-2-β_2_m^-/-^/IAβ^-/-^) 25 days after the subcutaneous injection of RAMOS cells. Fig 2A (left) and B (left) show HUMAMICE; while Fig 2A (right) and B (right) show Sure-L1 mice.

**Fig 3:**
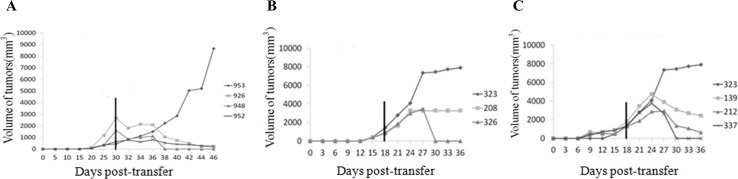
The tumor growth kinetics in HUMAMICE after transfer of RAMOS specific splenocytes from parental Sure-L1 mice. (A) Tumor growth kinetics following transfer of tumor specific splenocytes from Sure-L1 mice in three immuno-deficient HUMAMICE bearing a solid RAMOS tumor (mouse No.926; 948; 952) and the control mouse No.953 without the transfer of tumor-bearing cells. (B) Tumor growth kinetics following transfer of purified tumor specific CD8^+^ T lymphocytes from Sure-L1 mice in two immuno-deficient HUMAMICE bearing a solid RAMOS tumor (mouse No.208 ; 326) and the control mouse No.323 without transfer of tumor-bearing cells. (C) Tumor growth kinetics following transfer of tumor specific splenocytes depleted of CD8^+^ T cells (purified CD8^-^ T lymphocytes) from Sure-L1 mice in three immuno-deficient HUMAMICE bearing a solid RAMOS tumor (mouse No.139; 212; 337) and the control mouse No.323 without transfer of tumor-bearing cells.

To confirm the immuno-deficiency status of HUMAMICE, we engrafted subcutaneously human RAMOS tumor cells (10^7^cells/mouse) into HUMAMICE and immuno-competent parental Sure-L1 mice. Fig 2A (left) shows the kinetics of RAMOS tumor growth in six HUMAMICE (HLA-A2^+/+^/DR1^+/+^/H-2-β_2_m^-/-^/IAβ^-/-^/Rag2^-/-^/IL2rγ^-/-^/Perf^-/-^) (mouse No.912; 926; 953; 952; 948; 950), whereas Fig 2A (right) shows the tumor size in five immuno-competent Sure-L1 mice (HLA-A2^+/+^/DR1^+/+^/H-2-β_2_m^-/-^/IAβ^-/-^). In addition, Fig 2B shows the RAMOS cells tumor subcutaneous development in six HUMAMICE and five Sure-L1 mice during 25 days post the injection. Overall, tumors were not able to grow in immuno-competent mice whereas they induced tumors in HUMAMICE. Furthermore, to verify that the inability of HUMAMICE to reject RAMOS cells is due to lack of the mouse-derived immune system, we transferred total splenocytes, purified CD8^+^ T lymphocytes or purified CD8^-^ T lymphocytes derived from the immuno-competent Sure-L1 mice which had rejected the RAMOS cells into HUMAMICE that developed a solid tumor. Results of the kinetics of tumor growth after the transfer of total splenocytes, purified CD8^+^ T lymphocytes or purified CD8^-^ T lymphocytes from immuno-competent tumor-primed Sure-L1 mice are shown in Fig 3A, 3B and 3C, respectively. In Fig 3A, three immuno-deficient HUMAMICE bearing a solid RAMOS tumor (mouse No.926; 948; 952) rejected completely their tumors 15 days after cell-transfer of total splenocytes from immuno-competent tumor-primed Sure-L1 mice, while the volume of the tumor continued to grow in mouse (No.953) which did not receive cell transfer.

Fig 3B shows the example of HUMAMICE (No.208 and No.326) which bore solid RAMOS tumors. After the transfer of CD8^+^ purified T lymphocytes from immuno-competent tumor-primed Sure-L1 mice, only mouse No.326 could reject completely the tumors in 12 days, whereas mouse No.208 could only stop the tumor growth at 6 days and stabilized the tumor size without total rejection, compared with control mouse No.323 which did not receive cell transfer and in which the tumor continued to grow. As shown in Fi 3C, after receiving purified CD8^-^ T lymphocytes, from immuno-competent tumor-primed Sure-L1 mice, HUMAMICE (No.139, 212 and 337) bearing a solid RAMOS tumor started to reject their tumors by reducing progressively their size from 6 days post-transfer, while the tumors continued to grow in control mouse No.323 that did not receive cell transfer. Altogether, these results confirm that HUMAMICE are severely immuno-deficient.

### hPBMC engraftment in HUMAMICE

**Fig 4:**
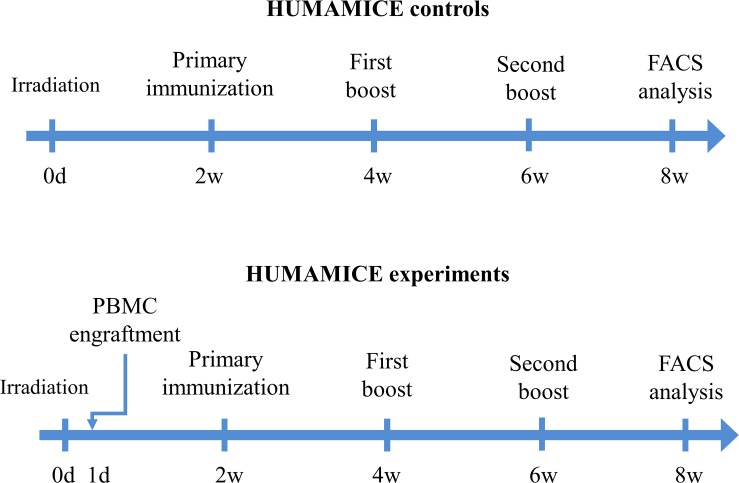
The experimental procedure of hPBMC transplantation and immunization with HBsAg vaccine in HUMAMICE.

In order to evaluate the engraftment of HLA-matching hPBMCs in this new immuno-deficient HLA-humanized mouse model, we transferred 10^7^ HLA-matched (HLA-A2^+^DR1^+^) hPBMCs intravenously into HUMAMICE after a total body irradiation and monitored the weight of mice and clinical GvHD signs once a week as described previously[18]. The experimental protocol is summarized in Fig 4.

**Fig 5:**
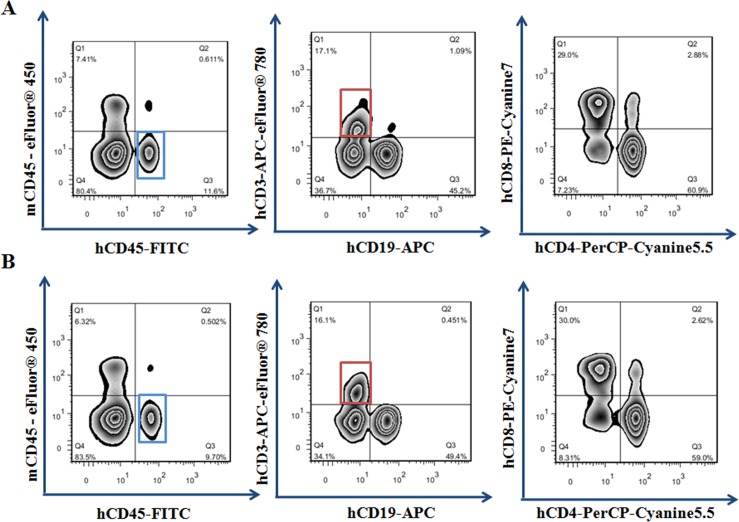
Flow cytometry analysis after five and eight weeks of hPBMC transplantation in HUMAMICE. Flow cytometry analysis was done at five, eight weeks and three months after the hPBMC were transferred. Results of two representative mice (from nine analyzed mice) which were analyzed at five and eight weeks after transplantation are shown in Fig 5. (A) For the mouse which was analyzed at 5 weeks after transplantation, 11.6% of splenocytes were composed by hCD45^+^ cells, with 45.2% of this population being hCD19^+^, and 17.1% being hCD45^+^hCD3^+^ T lymphocytes: 29.0% of them were hCD3^+^CD8^+^ and 60.9% were hCD3^+^CD4^+^ T cells. (B) For the mouse which was analyzed at 8 weeks after transplantation, 9.7% of splenocytes were composed by hCD45^+^ cells, with 49.4% of this population being hCD19^+^, and 16.1% being T lymphocytes: 30.0% of them were hCD3^+^CD8^+^ and 59.0% were hCD3^+^CD4^+^ T lymphocytes.

**Fig 6:**
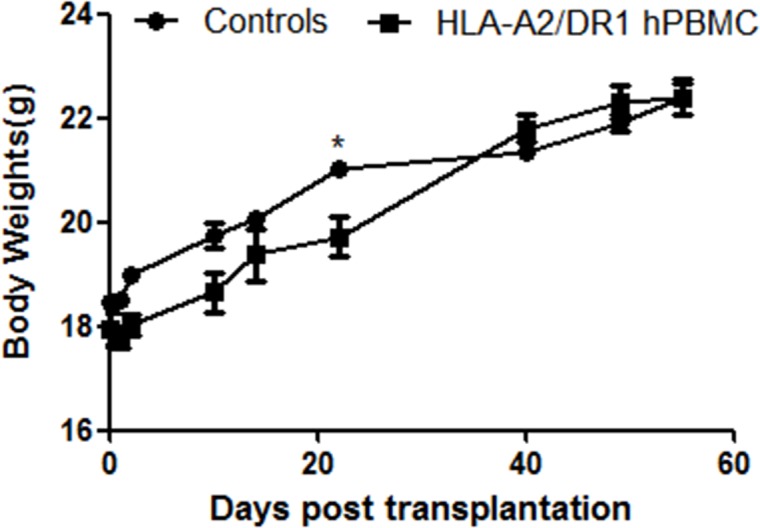
The evaluation of body weight of HUMAMICE after hPBMC transplantation. The average weight is shown as a percentage of starting weight. None of the HUMAMICE developed weight loss after hPBMC transplantation.

Flow cytometry analysis was done at five, eight weeks and three months after the hPBMC were transferred (Shown in Table 2). Results of two representative mice (from nine analyzed mice) which were analyzed at five and eight weeks after transplantation are shown in Fig 5. For the mouse that was analyzed at 5 weeks after transplantation (Fig 5A), 11.6% of splenocytes were composed by human CD45^+^ cells, with 45.2% of this population being hCD19^+^, and 17.1% of this population being hCD45^+^hCD3^+^ T lymphocytes: 29.0% of them were hCD8^+^ T lymphocytes (hCD45^+^hCD3^+^hCD8^+^) and approximately 60.9% were hCD4^+^ T lymphocytes (hCD45^+^hCD3^+^hCD4^+^). For the mouse which was analyzed at 8 weeks after transplantation (Fig 5B), 9.7% of splenocytes were composed by hCD45^+^ cells, with 49.4% of this population being hCD19^+^ B cells, and 16.1% of this population being hCD45^+^hCD3^+^ T lymphocytes: 30.0% of them were hCD8^+^ T lymphocytes (hCD45^+^hCD3^+^hCD8^+^) and approximately 59.0% were hCD4^+^ T lymphocytes (hCD45^+^hCD3^+^hCD4^+^). The constitutions of splenocytes of hPBMC-transplanted HUMAMICE were shown in Table 2. Moreover, none of the HUMAMICE developed GvHD signs or weight loss (shown in Fig 6).

**Table 2:** Summary of all the HUMAMICE transplanted with HLA-A2^+^/DR1^+^ hPBMCs.

Donor A/mouse	mCD45^-^hCD45^+^	hCD45^+^hCD19^+^	hCD45^+^hCD3^+^	hCD3^+^hCD4^+^	hCD3^+^hCD8^+^
Mouse 1 (5 weeks)	11.6%	45.2%	17.1%	60.9%	29.0%
Mouse 2 (5 weeks)	13.4%	51.9%	15.4%	68.3%	24.6%
Mouse 3 (5 weeks)	15.2%	56.7%	18.9%	52.4%	22.8%
Mouse 4 (8 weeks)	9.7%	49.4%	16.1%	59.0%	30.0%
Mouse 5 (8 weeks)	7.1%	60.8%	13.2%	40.9%	15.8%
Donor B/Mouse					
Mouse 6 (5 weeks)	19.2%	69.2%	14.7%	63.1%	20.9%
Mouse 7 (5 weeks)	15.8%	54.2%	15.1%	60.7%	19.2%
Mouse 8 (8 months)	8.9%	51.2%	20.7%	40.9%	12.1%
Mouse 9 (12 weeks)	5.4%	42.1%	12.2%	19.5%	4.5%

### The humoral immune response of HUMAMICE to a specific HBsAg vaccine

**Fig 7:**
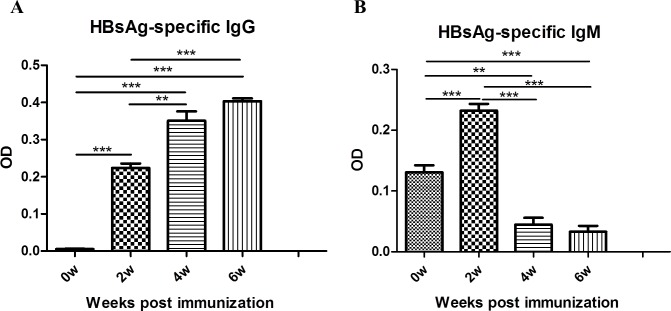
The humoral immune response of hPBMC-HUMAMICE after immunization with HBsAg vaccine. (A) The HBsAg specific IgG and (B) HBsAg specific IgM responses of immunized hPBMC-HUMAMICE before (0 w) and every two weeks after immunization (before each immunization boost).

We next evaluated the functional ability of these adoptively transferred HLA-matched-hPBMCs in HUMAMICE to respond to a specific antigenic stimulus, namely HBsAg vaccine. The schedule of immunization with the HBsAg vaccine is shown in Fig 4 and included a primary immunization two weeks after hPBMC transfer, followed by two immunization boosts every two weeks after primary immunization. The collected sera were analyzed with an HBsAg specific ELISA test according to the protocol described in Material and Methods. The background noise was represented by the mean of two HUMAMICE controls without transfer of hPBMCs. Fig 7A and 7B showed the HBsAg-specific-hIgG and HBsAg-specific-hIgM responses of immunized hPBMC-HUMAMICE before (0 w) and every two weeks after immunization (before each immunization boost). We observed a significant HBsAg-specific hIgG response which was amplified after recall immunization boots. And HBsAg-specific-IgM responses were detected after the primary-immunization, while no more responses were observed after recall immunization.

## Discussion

The development of small animal models that could mimic human immune responses is important to evaluate novel human useful clinical drugs or test new therapeutic treatments or new vaccines. In this paper, we show that HUMAMICE developed by backcrossing derived Sure-L1 mice with Rag2^-/-^ mice, IL2rγ^-/-^ mice and Perf^-/-^ mice can be efficiently reconstitute with HLA-matched PBMCs without developing GvHD and with a functional immune system able to launch a robust human T cell-dependent response. Human adaptive immune responses are HLA-restricted, implying that maintaining HLA compatibility is a requirement for normal human immune responses. Currently, no experimental preclinical mouse model can meet this requirement, including recently developed models such NSG[19], NOG or NRG mice. Thus, after hPBMC transferred, a rapid onset of xenogeneic Graft-*versus*-Host Disease (xeno-GvHD) is observed in all hPBMC-NSG mice. These mice exhibited weight loss, diarrhea, alopecia and pathological infiltration of activated human lymphocytes in liver, skin, or intestine, leading finally to death after 20 to 25 days[20]. It is interesting to note that the removal of murine MHC class I activity greatly reduces human *in vitro* T cell proliferative responses to murine cells and should thus lead to reduced xenogeneic GvHD following engraftment with human PBMC[21]. After HLA-matched hPBMC transferred, our mice did not develop any evidence of GvHD during the three month follow up period.

In present study, we developed a new improved immuno-deficient HUMAMICE model (HLA-A2^+/+^/DR1^+/+^/H-2-β_2_m^-/-^/IAβ^-/-^/Rag2^-/-^/IL2rγ^-/-^/Perf^-/-^) which could satisfy this requirement.

Indeed, the HUMAMICE strain is an immuno-deficient strain with a humanized immune microenvironment expressing both HLA class I and class II molecules, and which overcomes the lack of central thymic and peripheral human T-cell maturation through selective interaction with HLA class I and II molecules. The HUMAMICE lack almost all essential murine immune cells, as similarly demonstrated in NSG, NOG or NRG mouse model. In particular, a significant reduction of NK cells could favor the engraftment of foreign cells.

The proliferation of RAMOS tumor cells in immuno-deficient HUMAMICE but not in the parental immuno-competent Sure-L1 mice, and the subsequent tumor elimination by the transfer of immune cells from RAMOS-rejected Sure-L1 mice, confirmed the severe immuno-deficiency status of HUMAMICE. Interestingly, HUMAMICE could also be used as a readout system–together with their parental Sure-L1 mice or Sure-L1-TGFP mice (where all T cells co-express the GFP protein) or Sure-L1-FoxP3-GFP mice (where T cells co-expressed FoxP3 protein and GFP protein as fusion protein) to dissect the contribution role of each cell population to the protective immune responses against foreign antigens like pathogens. The present data indicated that all immune cells including T cells (CD3^+^CD4^+^, CD3^+^CD8^+^), B cells, and NK cells were involved in rejection of the RAMOS tumor.

After transferring 10^7^ HLA-matched (HLA-A2^+^DR1^+^) hPBMC cells intravenously into HUMAMICE, we found hCD45^+^ cells in splenocytes of HUMAMICE, which included both human B cells (hCD45^+^hCD19^+^) and human T cells (hCD45^+^hCD3^+^hCD4^+^ and hCD45^+^hCD3^+^hCD8^+^). More importantly, none of HUMAMICE developed any GvHD signs or weight loss after HLA-A2^+^DR1^+^ hPBMC were transferred.

Because of limited availability HLA-A2^+^DR1^+^ PBMCs from two donors, we could only perform one analysis in one mouse at 3 months after transfer of human PBMCs. At this time point, we detected around 42% hCD45^+^hCD19^+^ compared to 12% hCD45^+^ hCD3^+^ cells. These B cells (42%) could optimally interact with both reconstituted hAPC and hCD4^+^ cells. In addition, they could also interact with mAPC which expressed HLA-A2 and DR1. This could explain how both HBsAg-specific hIgG and hIgM antibodies are induced in our model. Our research indicated successful engraftment of hPBMCs in this new immuno-deficient HLA-humanized mouse model-HUMAMICE.

In the model of Xeno-GvHD, after intravenous transfer of hPBMCs in RAG2^-/-^c^-/-^ mice[22], high engraftment rates of human cells had been reported between 2 to 4 weeks after transplantation, with a T-cell chimerism of at least 20–48% in more than 90% of mice. This high engraftment rate was associated with the development of acute Xeno-GvHD and a mortality rate of 85% of the mice within 2 months. Interestingly only a small population of B cells (around 7–11%) residing in lymphoid compartments were detected in these mice explaining the detection of elevated plasma levels of IgG and IgM. In fact, it has also been reported that immunoglobulin production in hPBMC-SCID models could be high despite low numbers of B cells and was generally used as an indication of good engraftment of the human cells[23, 24, 25].

## Conclusions

The HUMAMICE model represents an improvement over the traditional immuno-deficient mice models like NSG and NOG mice, which have drawbacks such as development of xeno-GVHD. The HLA-compatible-hPBMC-HUMAMICE is thus a unique model that could be used to explore human immunology in a normal physiological situation (without xeno-GvHD) and could be useful for evaluation of cell-therapies, generation of human monoclonal antibodies, and production of novel vaccines.

Nonetheless, it should be noted that the new Humamice mice were made on the C57BL/6 genetic background, and then backcrossed for 7 generations, and not originally on the NOD background. The genetic background of the mice will impact the ability of the mice to sustain long-term engraftment of human HSC and PBMC in several immunodeficient strains. It was previously shown that NOD-scid mice supported higher levels of engraftment with human PBMCs than other strains tested, including C3H/HeJ-scid and C57BL/6-scid mice[26, 27].

More recently, it was shown that the NSG (NOD-scid IL2rγ^null^) mouse model supports higher levels of human hemato-lymphoid engraftment than BALB/c-Rag1^null^ IL2rγ^null^ mice[28], and that NOD-Rag1^null^ IL2rγ^null^ mice also engraft with human PBMC at higher levels than NOD-scid IL2rγ^null^ mice[29]. These data indicate that the difference of human PBMC engraftment between NOD-scid IL2rγ^null^ and BALB/c-Rag2^null^ IL2rγ^null^ mice is not due to scid vs. Rag1^null^ or Rag2^null^ genes but rather to genetic polymorphic background modifiers.

Genomic studies showed that these background modifiers are localized to the Idd13 locus, which encodes the Sirp gene. SIRP is mainly expressed by macrophages, DCs, and neutrophils, and its ligand, CD47, is expressed almost ubiquitously. The SIRP–CD47 interaction is involved in regulating macrophage-mediated phagocytosis by delivering a licensing signal to macrophages[30]. The NOD SIRP-α protein is more similar to the human SIRP-α protein than the C57BL/6 SIRP-α protein.It shows enhanced binding to the human CD47 ligand, and its expression on mouse macrophages leads to better support of human hematopoiesis[31]. The role of murine macrophages in the rejection of human cells and prevention of human reconstitution was confirmed when more successful engraftment rates of human cells were observed in neonate recipients that had lower macrophage activity and in adult murine hosts after depletion of macrophages following intraperitoneal injection of clodronate[32].

Moreover, NSG mice engraft with human PBMC better than H2d-Rag2^null^ IL2rγ^null^ mice. The production of NSG mice provides an immunodeficient mouse model lacking host NK cells, facilitating human PBMC engraftment. NSG mice support survival of engrafted human T cells, but the human cells mount a severe xenogeneic GvHD following engraftment into NK cell-deficient hosts.

In the present study, we made immuno-deficient HUMAMICE mice, which are mouse MHC Class I and Class II deficients, and express both HLA-A2 and HLA-DR1. It is interesting to note that the removal of mouse MHC class I activity greatly reduces human in vitro T cell proliferative responses to murine cells[33] and should lead to reduced xenogeneic GVHD following engraftment with human PBMC. This hypothesis was supported by experiments where PBMC were injected intravenously into NOD-scid β2m^null^ mice via the tail vein, resulting in transient engraftment and failing to induce xenogeneic GvHD[34]. Furthermore, it was previously shown that NOD-scid β_2_m^null^ mice support higher levels of human PBMC engraftment than NOD-scid mice[35], because in the absence of β_2_m, natural killer (NK) cell numbers and activity are severely depressed due to the lack of MHC class I expression[35].More importantly, the absence of β_2_m in host cells reduces xenogeneic GVHD responses of the human T cells. In addition, macrophages in HUMAMICE express both HLA-A2 and HLA-DR1, which are compatible with grafted human PBMC. In this HLA-matched situation, there are no allo-like or xeno-like rejection reactions, so the non-optimal mSIRP–hCD47 interaction does not affect long-term PBMC engraftment. However, it must be noted that HUMAMICE is not a good model for engraftment of non-HLA-matched PBMC or any human stem cells, including human HSCs, due to the NOD genetic background of the HUMAMICE and the lack of supporting cytokines, cytokines, supporting cells such as stromal cells, and human APCs in the mice.

In conclusion, despite some of the drawbacks listed above, the HLA-matched-hPBMC-HUMAMICE model allows the possibility of studying normal human immune responses in various human diseases, including infectious diseases and cancer in the HLA-matched population. It also represents a promising model for facilitating the development of novel vaccines and cellular therapies.
